# Cost-Effectiveness of MR-Mammography in Breast Cancer Screening of Women With Extremely Dense Breasts After Two Rounds of Screening

**DOI:** 10.3389/fonc.2021.724543

**Published:** 2021-09-09

**Authors:** Fabian Tollens, Pascal A. T. Baltzer, Matthias Dietzel, Moritz L. Schnitzer, Wolfgang G. Kunz, Johann Rink, Johannes Rübenthaler, Matthias F. Froelich, Clemens G. Kaiser

**Affiliations:** ^1^Department of Radiology and Nuclear Medicine, University Medical Centre Mannheim, Medical Faculty Mannheim-University of Heidelberg, Mannheim, Germany; ^2^Department of Biomedical Imaging and Image-Guided Therapy, Vienna General Hospital, Medical University of Vienna, Vienna, Austria; ^3^Department of Radiology, Friedrich-Alexander-University Hospital Erlangen, Erlangen, Germany; ^4^Department of Radiology, University Hospital, Ludwig Maximilian University of Munich, Munich, Germany

**Keywords:** breast MRI, MR-mammography, breast cancer, intermediate-risk screening, cost-effectiveness analyses, cost-effectiveness threshold

## Abstract

**Objectives:**

To evaluate the cost-effectiveness of MR-mammography (MRM) vs. x-ray based mammography (XM) in two-yearly screening women of intermediate risk for breast cancer in the light of recent literature.

**Methods:**

Decision analysis and Markov modelling were used to compare cumulative costs (in US-$) and outcomes (in QALYs) of MRM vs. XM over the model runtime of 20 years. The perspective of the U.S. healthcare system was selected. Incremental cost-effectiveness ratios (ICER) were calculated and related to a willingness to pay-threshold of $ 100,000 per QALY in order to evaluate the cost-effectiveness. Deterministic and probabilistic sensitivity analyses were conducted to test the impact of variations of the input parameters. In particular, variations of the rate of false positive findings beyond the first screening round and their impact on cost-effectiveness were assessed.

**Results:**

Breast cancer screening with MRM resulted in increased costs and superior effectiveness. Cumulative average costs of $ 6,081 per woman and cumulative effects of 15.12 QALYs were determined for MRM, whereas screening with XM resulted in costs of $ 5,810 and 15.10 QALYs, resulting in an ICER of $ 13,493 per QALY gained. When the specificity of MRM in the second and subsequent screening rounds was varied from 92% to 99%, the ICER resulted in a range from $ 38,849 to $ 5,062 per QALY.

**Conclusions:**

Based on most recent data on the diagnostic performance beyond the first screening round, MRM may remain the economically preferable alternative in screening women of intermediate risk for breast cancer due to their dense breast tissue.

## 1 Introduction

In line with current recommendations, MR-mammography (MRM) has been clinically accepted for various indications such as screening women at high risk for breast cancer, diagnostic evaluation in cancer of unknown primary, and as a problem solver in special cases (1, 2).

Recent data has hinted towards a role of MRM in an extended set of indications, such as screening women at intermediate risk of breast cancer due to their elevated density of breast tissue. The superior diagnostic performance of MRM compared to x-ray based techniques has been demonstrated in several prospective multicenter trials (3–5). Data on the first screening round of the DENSE trial indicated an incremental cost-effectiveness ratio (ICER) of two-yearly MRM screening below $ 10,000 per QALY gained as previously published in our model-based cost-effectiveness analysis (6).

Based on current data, the Dutch parliament has lately decided to introduce MRM as a screening tool for women of intermediate risk due to their breast density as the first country to recommend so (7).

Cost-effectiveness analyses have become a widely accepted methodology in order to direct healthcare resource allocation (8). In particular, expensive innovative medical procedures, diagnostic tests and screening programs are inherently afflicted with ongoing claims not only to provide proof of favorable downstream effects but also superior economic value (9). Therefore, modern concepts of economic studies are increasingly being applied to evaluate diagnostic strategies in screening.

The economic potential of MR-based techniques in breast cancer screening has been demonstrated in various collectives - all with a favorable economic outcome compared to x-ray based techniques (6, 10–13). However, the definition of valid input parameters for these model-based analyses depends on representative real-world data. Long-term data on screening women with intermediate risk for breast cancer with MRM due to their elevated breast density has been unavailable up to this point.

Most recently, Veenhuizen et al. closed this gap by delivering unprecedented data on the diagnostic performance of MRM in breast cancer screening beyond the first screening round (5). A reduced incremental cancer detection rate of 5.8 per 1000 screening examinations in the second screening round (respective 16.5 in the first screening interval) as well as an increased specificity of 97% for the second screening round (respective 92% in the first screening round) were observed in the second screening round of the DENSE trial. All breast cancer cases were detected in an early stage (stage 0-1) and were node negative (5), underlining the diagnostic and prognostic potential of MRM, and hinting towards its economic value beyond the first screening interval.

As outlined in our previous cost-effectiveness analyses, an optimal specificity as well as a minimal rate of false positive findings is required to economically justify MRM as a screening tool in patients of intermediate risk for breast cancer.

Therefore, this study seeks to evaluate the cost-effectiveness of MRM in comparison to x-ray mammography (XM) in screening women of intermediate risk for breast cancer due to their elevated breast density, considering the changes in specificity and false positives in a follow-up situation.

## 2 Materials and Methods

### 2.1 Economic Modelling

#### 2.1.1 Decision Model

**Figure 1:**
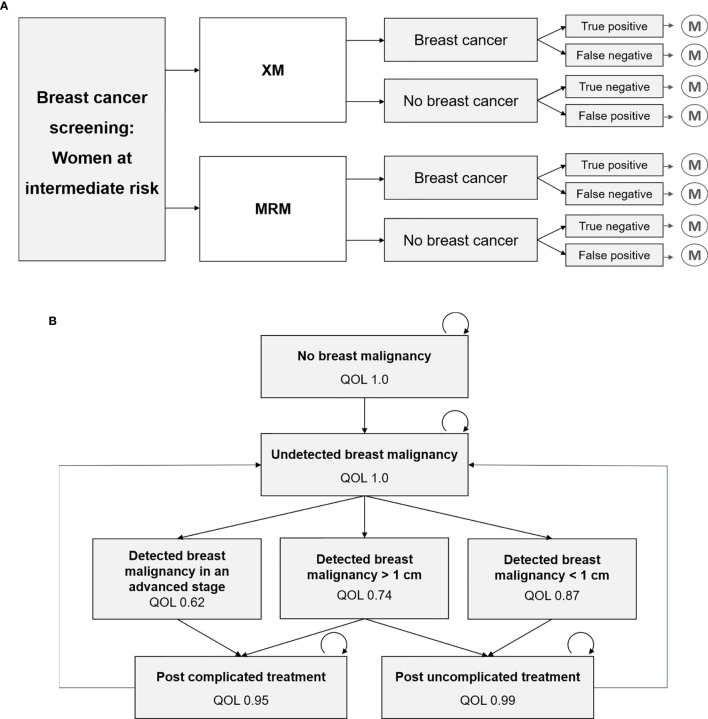
Decision tree and Markov model, which have recently been developed and refined for this study (6, 12, 13). **(A)** Decision model that represents the diagnostic strategies x-ray based mammography (XM) versus MR-mammography (MRM), and the respective outcomes true positive, false negative, true negative and false positive, that each result in a Markov model simulation. **(B)** Markov model with various health states and their associated quality of life (QOL). Transition to death is not depicted.

To compare the diagnostic strategies XM versus MRM in a screening setting, a decision model was designed that included the diagnostic outcomes true positive, true negative, false positive and false negative (**Figure 1A**), based on previously published cost-effectiveness analyses (6, 12, 13).

#### 2.1.2 Markov Model

A recently developed Markov Model for breast cancer screening was adapted for the two-yearly screening of women at intermediate risk of breast cancer (**Figure 1B**). Intermediate risk was defined by elevated breast tissue density, which is further specified in 2.2.1. Long-term costs and outcomes were simulated using a cycle-length of one year and a total model runtime of 20 years. Quality of life in each Markov state was used to calculate cumulative quality-adjusted life years (QALYs) for each diagnostic strategy. Correspondingly, annual costs were assigned to each Markov state and summed up over the total duration of 20 years. In line with current recommendations (1, 2, 14), a screening interval of two years was assumed.

### 2.2 Input Parameters

Input parameters were extracted from recent literature (**Table 1**) closely following international standards on the conduct and methodological practice of cost-effectiveness analyses (16, 31).

**Table 1:** Model input parameters for the economic modelling, that have recently been published and adapted for this analysis (6, 12, 13).

Variable	Estimation	Source
Pre-test probability of malignant lesion	1.65%	(4)
Starting age of Markov simulation	55	(4)
Screening interval	two years	
Incidence of breast cancer	Age-specific incidence rates	SEER age-adjusted incidence rates 2017 (15)
Assumed WTP	$ 100,000	(16)
Discount rate	3.00%	(16)
**Diagnostic test performances**		
Sensitivity of XM	41.2%	(17–21)
Specificity of XM	90.0%	(22)
Sensitivity of MRM	95.2%	(4)
Specificity of MRM, first screening round	92.0%	(4)
Specificity of MRM, subsequent screening rounds	97.0%	(5)
Biopsy rate among false positives, first screening round	67.2%	(4)
Biopsy rate among false positives, second screening round	80.2%	(5)
**Costs**		
Cost of XM	$ 101.52	Medicare (G0202)
Cost of full-scale MRM	$ 314.00	Medicare (CPT code 77047)
No further action (true negative)	$ 0.00	Assumption
Biopsy	$ 1,536.00	Medicare (CPT code 19083)
Cost of treatment for tumor < 1 cm	$ 60,637	(23)
Cost of treatment for tumor > 1 cm	$ 82,121	(23)
Cost of treatment for advanced stage breast malignancy	$ 129,387	(23)
**Utilities**		
QOL of patients without detected tumor	1.00	Assumption
QOL of patients with detected tumor < 1 cm	0.87	(24)
QOL of patients with detected tumor > 1 cm	0.74	(25)
QOL of patients with detected regional breast cancer in an advanced stage	0.62	(26)
QOL of patients post simple treatment	0.99	Assumption
QOL of patients post intensive treatment	0.95	Assumption
Reduction in QOL due to false positive finding	0.01	Assumption
Death	0.00	Assumption
**Transition probabilities**		
Risk of death without tumor (yearly)	age adjusted	US Life Tables 2017, women of all ethnicities (27)
Risk of death with undetected tumor	10.00% in 10 years	Assumption
Risk of death with detected < 1 cm tumor	0.11%	(28)
Risk of death with detected > 1 cm tumor	0.78%	(28)
Risk of death with detected tumor in advanced stage	1.81%	(28)
Probability of initial R0 resection < 1 cm	100.00%	Assumption
Probability of initial R0 resection ≥ 1 cm	90.00%	(29)
Proportion of N+ in < 1 cm tumors	0.00%	Assumption
Proportion of N+ in > 1 cm tumors	40.00%	(30)
Proportion of successfully treated tumors < 1 cm if detected within 1 screening interval	100.00%	Assumption

CPT, current procedural terminology; MRM, MR-mammography; QOL, quality of life; SEER, surveillance, epidemiology, and end results; WTP, willingness to pay; XM, x-ray mammography.

#### 2.2.1 Screening Collective

Women at intermediate risk of breast cancer due to their elevated breast density (ACR BI-RADS category 4 or D) represented the study collective, with a mean age of 55 years at the beginning of the screening program as reported by Bakker et al. (4).

#### 2.2.2 Diagnostic Performance Parameters

The diagnostic accuracy of the modalities were extracted from literature (4, 5, 17–22). Importantly, the specificity of MRM has been demonstrated to rise from 92% in the first screening round to 97% in the second round, which was assumed to stay constant in subsequent screening rounds.

#### 2.2.3 Quality of Life

Quality of life was estimated for each Markov state based on published literature (24–26). Size of the tumor at the time of detection, stage of disease and the respective therapy affected the patients’ quality of life.

#### 2.2.4 Cost Estimates

Both short- and long-term costs in US-$ were included (**Table 1**) in order to calculate cumulative total costs of each diagnostic strategy based on Medicare Current Procedural Terminology (CPT) codes and literature (23). The perspective of the U.S. healthcare system was selected to estimate costs due to standardization and comparability of the data. Costs for false positive results were simulated throughout the entire time span. False positive findings of XM resulted in a biopsy, whereas MRM resulted either in a follow-up examination or a biopsy (**Table 1**).

#### 2.2.5 Transition Probabilities

Age-adjusted incidence rates were collected from the Surveillance, Epidemiology, and End Results (SEER) Program (15). Disease-specific risk of death was estimated based on the NHS Predict model (28). General age-adjusted death rates were extracted from the U.S. Life Tables (27). Resection status and nodal status were estimated depending on the stage of disease (29, 30).

### 2.3 Analysis

#### 2.3.1 Cost-Effectiveness Analysis

The U.S. healthcare system was chosen as the study perspective. Outcomes were modelled by calculating the average cumulative QALYs for each diagnostic strategy, costs were measured in US-$. Dedicated software for economic modelling and decision analysis was used to carry out Markov modelling and cost-effectiveness analyses (TreeAge Pro 2020, TreeAge Software, Williamstown, MA). According to recommendations on the conduct of cost-effectiveness analyses, an annual discount rate of 3% was applied both for costs and outcomes (16). The willingness to pay (WTP)-threshold was set to $ 100,00 per QALY gained (32, 33), so that an incremental cost-effectiveness ratio (ICER) below this value indicated favorable cost-effectiveness.

#### 2.3.2 Sensitivity Analysis

**Figure 2:**
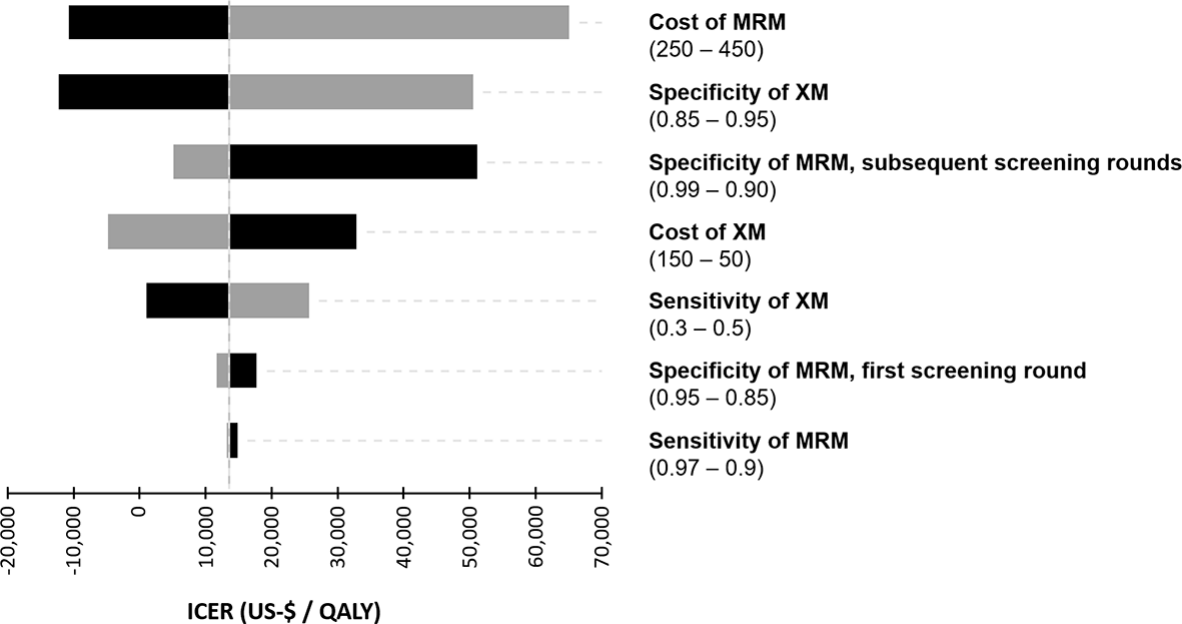
Tornado diagram of the deterministic sensitivity analysis. Costs of the diagnostic procedures (US-$) and the diagnostic performance were varied within a reasonable range to illustrate their impact on the incremental cost-effectiveness ratio (ICER).

**Figure 3:**
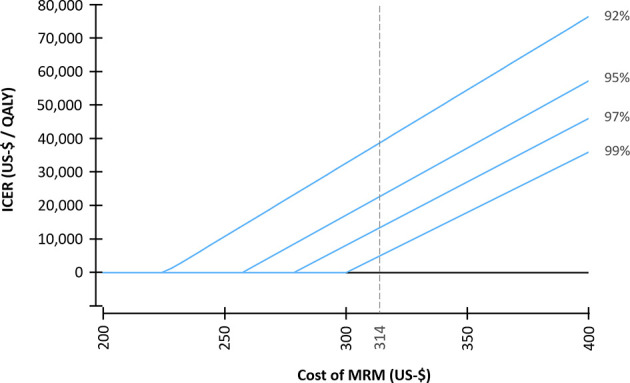
Incremental cost-effectiveness ratio (ICER) for varying costs of MR-mammography (MRM). A specificity of MRM of 92% was selected for the first screening round. For the subsequent screening rounds, varying specificities (92% - 99%) were assumed. An average cost per examination of $ 314 was assumed for MRM in the base case scenario.

The diagnostic performance and the cost of the screening modalities were varied in a deterministic sensitivity analysis and the resulting ICER was simulated in order to examine the impact of variations in the input parameters (**Figure 2**). In the next step, the relationship between varying costs of MRM and the specificity of MRM in the second and subsequent screening rounds and their impact on the resulting cost-effectiveness were modelled (**Figure 3**).

**Figure 4:**
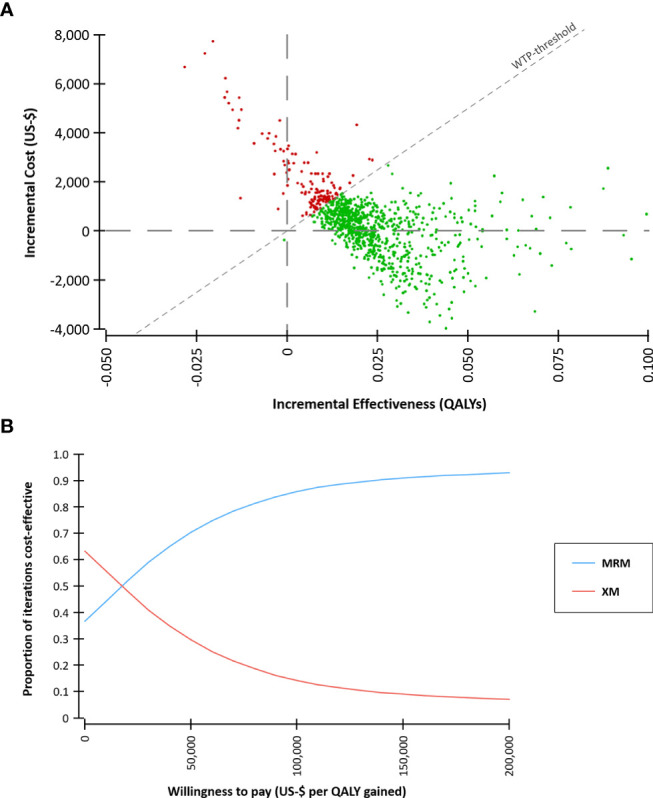
Probabilistic sensitivity analysis based on 30,000 Monte Carlo simulations. **(A)** Incremental costs and effects comparing MR-mammography (MRM) versus x-ray mammography (XM). A willingness-to-pay (WTP) threshold of $ 100,000 per quality-adjusted life year (QALY) gained was assumed. **(B)** Cost-effectiveness acceptability curve. At a WTP of $ 100,000 per QALY gained, 86% of the iterations were cost-effective.

A probabilistic sensitivity analysis with 30,000 Monte Carlo iterations was conducted to reflect the uncertainty of the input parameters and a cost-effectiveness acceptability curve was calculated (**Figure 4**).

## 3 Results

### 3.1 Cost-Effectiveness Analysis

In the base case scenario, applying MRM in the screening of women with intermediate risk for breast cancer resulted in more QALYs, i.e. favorable effects, than XM-based screening, but was associated with higher costs. Over the time frame of 20 years, the strategy MRM resulted in average cumulative costs of $ 6,081 per woman and average cumulative effects of 15.12 QALYs, whereas screening with XM resulted in costs of $ 5,810 and 15.10 QALYs (**Table 2**). The resulting ICER was $ 13,493 per QALY gained.

**Table 2:** Cost-effectiveness analysis of the base-case scenario comparing MR-mammography (MRM) to x-ray mammography (XM).

Strategy	Cumulative discounted costs (US-$)	Incremental costs (US-$)	Cumulative discounted effects (QALYs)	Incremental effects (QALYs)	Incremental cost-effectiveness ratio (US-$/QALY)
**XM**	5,810	–	15.099	–	
**MRM**	6,081	271	15.120	0.020	13,493

The cumulative discounted costs (US-$) and outcomes (quality-adjusted life years, QALYs) were calculated for a time frame of 20 years.

The specificity of MRM was set to 92% in the first screening round. When varying the specificity for the subsequent screening rounds from 92% to 99%, the ICER resulted in a range from $ 38,849 to $ 5,062 per QALY gained (**Table 3**).

**Table 3:** Cost-effectiveness of MR-mammography (MRM) for varying specificities of MRM in the second and the following screening rounds, with cumulative costs and effects and the incremental cost-effectiveness ratio compared to x-ray mammography.

Specificity of MRM in subsequent screening rounds	Cumulative discounted costs (US-$)	Cumulative discounted effects (QALYs)	Incremental cost-effectiveness ratio (US-$/QALY)
**92%**	6,492	15.117	38,849
**93%**	6,410	15.118	33,206
**94%**	6,328	15.118	27,873
**95%**	6,246	15.119	22,824
**96%**	6,163	15.119	18,037
**97%**	6,081	15.120	13,493
**98%**	5,999	15.120	9,173
**99%**	5,917	15.121	5,062

### 3.2 Sensitivity Analysis

#### 3.2.1 Deterministic Sensitivity Analysis

In a one-way sensitivity analysis, which is illustrated in a tornado chart (**Figure 2**), the cost of MRM was identified as the most important driver of cost-effectiveness. The specificity of XM and MRM were also key determinants of the ICER. Interestingly, the sensitivity of MRM did not show a significant impact on cost-effectiveness in the chosen model.

Therefore, the impact of the cost of MRM and the specificity of MRM in subsequent screening rounds on the ICER was further investigated in a deterministic sensitivity analysis (**Figure 3**). Assuming a specificity in subsequent screening rounds of 99%, a cost per examination of MRM of $ 296 resulted in equal costs of the MRM- and XM-strategy (ICER = 0); whereas for a specificity of 92%, a cost of MRM of $ 224 resulted in equal costs of both strategies.

#### 3.2.2 Probabilistic Sensitivity Analysis

In the Monte Carlo simulation, the majority of iterations were below a willingness-to-pay threshold of $ 100,000 per QALY gained (**Figure 4**). 37% of the iterations were characterized by lower costs of the MRM-strategy compared to the XM-strategy. This indicates that screening with MRM might be less costly than with XM in a part of the screening collective. The cost-effectiveness acceptability curve demonstrates the stability of the findings across a range of WTP-thresholds. At a WTP of $ 100,000 per QALY gained, 86% of the iterations were cost-effective.

## 4 Discussion

The new data by Veenhuizen et al. for the first time allow for a cost-effectiveness analysis addressing the diagnostic shifts between screening rounds (5).

In our study, we were able to reconfirm the cost-effectiveness of MRM as a screening tool for patients of intermediate risk for breast cancer, considering the shift in diagnostic performance over the course of two screening rounds.

In the second screening round of the DENSE trial (incidence round), a decreased cancer detection rate was observed due to filtering effects of the first round (prevalence round) while all newly detected breast cancer cases were in an early stage (T1). Thus, the balance of the costs of screening vs. the number of detected cases of breast cancer could be shown to be impaired in the second screening round and the cost per detected case of breast cancer was higher than in the first screening round. On the contrary, improvements in specificity, reduced false positive findings and hence smaller follow-up costs counteract these effects. An improved specificity as well as reduced rate of false positive findings apparently outweighed the decreased cancer detection rate in follow-up examinations. Our results identified ICER levels of around $ 13,493 per QALY gained (respective ICER $ 38,849 per QALY in the first screening round).

Naturally, modelling the cumulative average costs and effects as conducted in this economic evaluation does not allow for a differentiation of these effects.

The reasons for the increased specificity may present a matter of scientific discussion. However, Veenhuizen et al. stressed the role of prior imaging from preceding screening rounds and their possible comparison in order to reduce false positive findings.

Decreased false positive findings in subsequent screening rounds offer the potential to reduce adverse effects of screening, e.g. unnecessary biopsies, and to further improve the cost-effectiveness of screening of women with intermediate risk of breast cancer.

The results of our study are generally in line with recent cost-effectiveness evaluations examining breast cancer screening with various imaging techniques. We have previously indicated an economical benefit of MRM in screening women at intermediate risk in westernized countries with their respective WTP levels, i.e. their accepted monetary thresholds for gains in quality-adjusted life years (6, 13). Compared to DBT, abbreviated breast MRI was cost-effective in screening women at intermediate risk, with an ICER below $ 20,807 per QALY gained (13).

### 4.1 Study Limitations

Markov models allow for a simplified simulation of successive disease states and associated costs, but can never accurately reflect any manifestation of clinical reality. For reasons of transparency and comparability of the findings, the U.S. healthcare system perspective was selected for this analysis, which enables comparisons to prior studies but may not be transferable to different contexts.

ICER-levels may be impacted slightly by the evolution of our Markov models over time, leading to some distortion of the effects. However, the authors believe that the development of the model contributes to a more realistic simulation of economic outcomes.

So far, only data on the first two screening rounds have been published from the prospective multi-centre trials on MR-based screening (3–5). Data on the long-term diagnostic performance, interval cancer rates and effects on mortality have been unavailable so far. Therefore, diagnostic performance parameters of the second screening round were extrapolated to subsequent screening rounds in our study.

In conclusion, updating our economic modelling by recently reported data on diagnostic performance beyond the first screening round, the superior cost-effectiveness of MRM in screening women with dense breast tissue for breast cancer could be reconfirmed. Improved specificity and reduced false positive findings in subsequent screening rounds appear to have more impact on cost-effectiveness than reduced cancer detection rates.

## Data Availability Statement

The original contributions presented in the study are included in the article/supplementary material. Further inquiries can be directed to the corresponding author.

## Author Contributions

FT, PB, MF, and CK contributed to conception and design of the study. FT and CK performed the economic modelling. MF, MS, WK, and JRu contributed to input data collection. FT and CK wrote the first draft of the manuscript. All authors contributed to the article and approved the submitted version.

## Conflict of Interest

The authors declare that the research was conducted in the absence of any commercial or financial relationships that could be construed as a potential conflict of interest.

## Publisher’s Note

All claims expressed in this article are solely those of the authors and do not necessarily represent those of their affiliated organizations, or those of the publisher, the editors and the reviewers. Any product that may be evaluated in this article, or claim that may be made by its manufacturer, is not guaranteed or endorsed by the publisher.
